# Development of an AI predictive model to categorize and predict online learning behaviors of students in Thailand

**DOI:** 10.1016/j.heliyon.2024.e32591

**Published:** 2024-06-06

**Authors:** Jira Chonraksuk, Surapon Boonlue

**Affiliations:** Faculty of Industrial Education and Technology, King Mongkut's University of Technology, Thailand

**Keywords:** AI predictive model, Online learning behavior, Thai MOOC, Online learning

## Abstract

This qualitative study has three objectives: (1) to develop a predictive AI model to categorize the online learning behavior of Thai students who study through a Thai Massive Open Online Course (MOOC); (2) to categorize students’ online behavior in a Thai MOOC; and (3) to evaluate the prediction accuracy of the developed predictive AI models. Data were collected from 8000 learners enrolled in the KMUTT015 course on the Thai MOOC platform. The k-means clustering algorithm classified learners enrolled in the Thai MOOC platform based on their online learning behaviors. The decision tree algorithm was used to assess the accuracy of the AI model prediction capability. The study finds the predictive AI model successfully categorizes learners based on their learning behaviors and predicts their future online learning behaviors in the online learning environment. The k-means clustering algorithm yields three groups of learners in the Thai MOOC platform: High Active Participants (HAP), Medium Active Participants (MAP), and Lurking participants. The findings also indicate high predictive accuracy rates for each behavioral group (HAP cluster = 0.98475, Lurking participants cluster = 0.967625, and MAP cluster = 0.955375), indicating the proficiency of the AI predictive model in forecasting learner behavior. The results of this study will benefit the design of online courses that respond to the needs of students with different online learning characteristics and help them achieve a high level of academic performance.

## Highlights

• A predictive AI model was developed to increase the efficiency of online learning in Thai MOOCs.
• AI model predicted online learning behavior with high accuracy, grouping learners into HAP, MAP, and LP using k-means clustering.
• This predictive AI model will help instructors tailor their online curricula to match diverse learners' characteristics and enhance their achievement.

## 1 Introduction

The emergence of online learning platforms has ushered in a transformative era in education, expanding opportunities for accessible and flexible learning experiences [1,2]. While these platforms have significantly broadened educational access, they have also introduced unique challenges, particularly in ensuring learner engagement, success, and retention. The fundamental concern plaguing online education is the substantial gap between the number of students enrolled in courses and the successful course completion rate, which generally hovers between 6 % and 13 % [3,4]. This has raised critical questions about the efficacy of online teaching methodologies and the need for personalized learning experiences tailored to diverse learner behaviors [[5], [6], [7]], influenced by factors such as individual aptitude, prior knowledge, and the learning environment [5,[8], [9], [10]]. Learners can exhibit various behaviors, from solid foundational knowledge to grappling with course content, varying degrees of time management skills, and differing abilities to navigate the online learning ecosystem [3,6,11].

Massive Open Online Courses (MOOCs) offer a model of online education characterized by unlimited enrollment and open web-based access. The pedagogical approach of these courses often centers on pre-recorded video lectures, readings, online assessments, and discussion forums [12]. Prominent MOOC providers include Coursera, Udacity, and EdX. The open-source Open EdX software has reached international prominence as a platform for MOOC development, powering systems like JMOOC, KMOOC, and XuetangX [[13], [14], [15]]. In Thailand, the Thai MOOC has been used as a platform for organizing online teaching in a national open system for the public since 2016 under the supervision of the Office of the Permanent Secretary, Ministry of Higher Education, Science, Research and Innovation, to expand educational opportunities for Thai people and raise the quality of online courses to international standards [1]. However, it was found that the average student completion rate compared to registration numbers was very low (12.6 %) since this platform allows students to enroll and plan their studies independently without supervision. Some students did not effectively manage their studies, while others registered for courses that did not match their needs. Furthermore, some students lacked the necessary background knowledge and could not understand the lesson, resulting in their dropping out [3].

Innovative solutions, such as predictive AI models, are needed to increase the efficiency of online learning through MOOCs [3]. An AI predictive model is created with the use of machine learning and deep learning capabilities to analyze historical and current data to make predictions on future outcomes [16]. These models leverage machine-learning techniques to analyze the vast number of learner interactions and behavior datasets in online learning environments. AI-driven solutions provide personalized learning experiences by identifying patterns, making data-driven predictions, and optimizing learner outcomes [4]. Today, many educators and academicians use AI technology to improve learning experiences by tailoring courses for students with different online learning behaviors. For instance Ref. [17] employed AI technology to analyze teaching strategies and increase the efficiency of online courses [18]. used AI to predict the dropout behaviors of students who left school and find timely solutions [19]. used AI to classify learners' behaviors from MOOC data to improve teaching styles and design courses to meet student needs. This evidence indicates the AI model's capability to analyze students' learning behavior to improve online learning experiences.

Although the predictive AI model's ability to categorize and predict students' learning behavior, has been acknowledged, AI has never been used to improve the efficiency of Thai MOOCs. Hence, this research aims to develop a predictive AI model and evaluate its accuracy. The insights produced by the model can support instructors in designing effective online courses and refining their teaching approaches, ultimately leading to improved rates of student success. The objectives are as follows: (1) develop a predictive AI model to categorize the online learning behavior of Thai students studying through a Thai MOOC, (2) categorize the online behavior of students enrolled in a Thai MOOC, and (3) evaluate the accuracy of the developed predictive AI models in forecasting learners' online behavior.

## 2 Related work

In the landscape of online learning and predictive AI models, we developed our research study based on prior research endeavors to improve understanding and enhance learner behavior prediction in online learning environments. This section reviews the contributions from the existing literature that inform and contextualize our study.

### 2.1 Grounded theories

Self-determined learning forms a critical aspect of online education, particularly in MOOCs. This refers to learners’ ability to take ownership of their learning process, set their own goals, and regulate their learning activities [[20], [21], [22]]. Self-determined learning is essential in MOOCs as it empowers learners to take control of their own education, set meaningful goals, and engage in activities that align with their interests and needs [[22], [23], [24]]. Adaptive learning, a methodology that focuses on customizing the learning experience to meet the unique needs of each individual learner, is also important [25]. In MOOCs, adaptive learning technologies use data and algorithms to assess the strengths, weaknesses, and learning style of each learner. This allows educational content to be tailored and delivered to those specific requirements [[26], [27], [28]]. This personalized approach can enhance self-determined learning by providing content and activities that align with individual goals and interests. Additionally, adaptive learning enables learners to set achievable goals through the provision of the appropriate level of challenge and support.

### 2.2 Predictive AI models for online learning

As the popularity of online learning continues to rise, so does the need for innovative solutions to enhance learner engagement, success, and retention. Predictive AI models have emerged as a promising avenue to address these challenges [3]. These models leverage machine-learning techniques to analyze the many learner interactions and behavior datasets in online learning environments. AI-driven solutions provide personalized learning experiences by identifying patterns, making data-driven predictions, and optimizing learner outcomes [4].

Predictive models in online education are not new. The methodology of this study aligns with approaches used in other studies. For example, similar studies have used decision trees to develop predictive AI models [[29], [30], [31]], whereas others have utilized k-means clustering for student classification and for informing the training of predictive models [32,33]. Researchers have explored various methodologies to predict learner outcomes, including dropout rates, course completion, and academic performance [[8], [9], [10], [11],^29^,^31^,[34], [35], [36], [37]]. These studies have often relied on learning analytics methods to extract valuable insights from the data in online learning environments [[8], [9], [10], [11],^29^,^31^,[34], [35], [36], [37]]. Applying machine-learning algorithms, such as k-means clustering and decision trees, has proven effective in identifying behavioral patterns and making predictions [2,6,7,9,11,36].

### 2.3 Learner behavior classification

Based on online learning behaviors and participation rate in the course activities [38], classified learners into three types: lurking, moderately active, and memorably active, based. These studies identified commonalities and differences among learners, mirroring our effort in Phase 1 to categorize learners into High Active Participants (HAP), Medium Active Participants (MAP), and Lurking participants (LP) using k-means clustering. This classification serves as the foundation for our predictive AI models.

One of the primary goals of predictive AI models in online learning environments is to enable personalized and adaptive learning experiences [5,10,38]. These models categorize learners into groups based on their behavior: the HAP, MAP, and LP groups [5,39]. Once categorized, learners receive tailored content and interventions that align with their needs and motivations [8,29,31,[34], [35], [36]] improving engagement, satisfaction, and learning outcomes.

While predictive AI models offer significant promise, they also raise ethical considerations, particularly regarding learner privacy and data security [1,8]. It is essential to ensure that AI-driven insights are used responsibly and transparently. Learner consent mechanisms and clear guidelines for data usage are critical components of ethical AI applications in education.

### 2.4 MOOC platforms

The Thai MOOC platform, derived from the Open Edx software, aligns with the broader MOOC trend. Research guides, such as the Edx Research Guide [40], have provided valuable insights into tracking student behavior in MOOCs. Our study borrows from these insights and adapts them to the specific context of the Thai MOOC platform [40,41].

Online courses hosted on the Thai MOOC platform predominantly use video to deliver content, constituting over 65 % of the total content [13]. In addition, they incorporate interactive elements such as quizzes (review form during class) and exams (evaluation form at the end of the lesson) [1,2,13]. Other content types, such as text, images, and discussion forums, may also be present. Consequently, the logs of online learning behavior data collected from learners are categorized into four primary types [1,13].

*Common Event Type*: This category entails fundamental behavioral information about learners’ interactions with online courses. Key data points include attendance records, academic performance checks, time spent studying, and success rates based on course registrations and completion. These metrics provide a foundational understanding of learner engagement and progress.

*Navigational Event Type*: Navigational events capture learners’ interactions with navigation buttons in online courses and web pages. These interactions include clicking forward or backward in a course module or exiting the online course learning screen. Analyzing these events offers insights into how learners navigate course content and their engagement patterns.

*Video Interaction Events Type*: Given the prominent use of video content, this category focuses on behavioral data related to learners’ interactions with video components. These interactions include video playback, skipping video content, adjusting playback speed, and completing video viewing. Understanding how learners engage with videos is crucial to providing insights into their multimedia learning preferences and behaviors.

*Problem Interaction Events Type*: This category entails learners' interactions with quizzes and exams throughout the learning period. Data collected include responses to quiz questions, exam completion, and any interactions related to assessments. Analyzing this data type sheds light on learners’ performance, comprehension, and assessment-related behaviors.

**Fig. 1:**
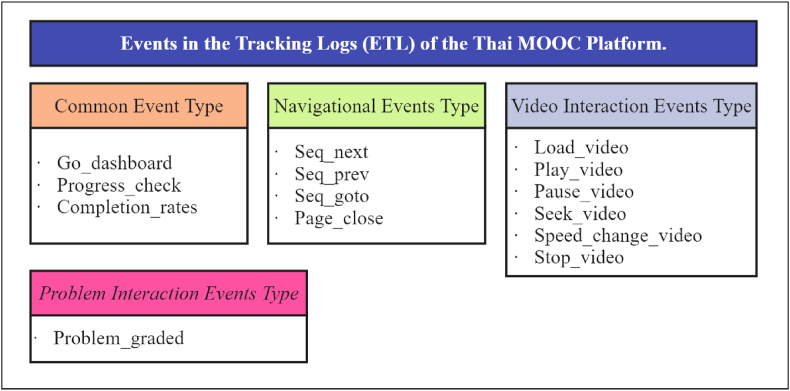
Events in the Tracking Logs (ETL) of the Thai MOOC platform.

Categorizing online learning behavior data into these four types, as shown in Fig. 1 provides insights into how learners engage with the online courses in the Thai MOOC platform. This multidimensional approach enables researchers to explore various facets of learner behavior, from foundational metrics to multimedia engagement, navigation patterns, and assessment performance. This rich dataset supports developing, evaluating, and enhancing predictive AI models for online education in the dynamic landscape of the Thai MOOC platform [40]. Thus, Fig. 1 provides a category name that lists insights in events in the tracking logs (ETL) of the Thai MOOC Platform, including common event, navigational event, video interaction events, and problem interaction events types.

## 3 Research methodology

This study employed a quantitative research method to obtain outcomes based on the research objectives. The research procedures are detailed below.

### 3.1 Data sources

This study's primary data source was the online learning behavior of 8000 learners enrolled in the KMUTT015 course on the Thai MOOC platform. The data analysis focuses on ETL (Fig. 1), with a log file size encompassing 4 categories and 14 variables, and the clustering process use k-means clustering; this course was selected because of its specific characteristics. The collected data includes a large dataset comprising learner interactions and the participants' high achievement rates.

Data collection was conducted over 11 months, from February 1, 2021, to December 30, 2021. This timeframe allowed for a comprehensive exploration of seasonal variations and long-term trends in online learning behavior. The primary data sources comprised ETL maintained by the Thai MOOC platform. These logs adhered to the data variables and tracking mechanisms outlined in the Open Edx system manual [40] This meticulous adherence to data variables ensured the accuracy and consistency of the dataset.

### 3.2 Instruments

Data preprocessing played a pivotal role in the research process. ELK (Elasticsearch, Logstash, Kibana), a versatile and robust data analysis system, was installed on a dedicated server hosted at elk.thaimooc.org to efficiently organize and structure the raw data. ELK served as the core instrument for data preprocessing, facilitating the conversion of raw data into a format suitable for in-depth analysis.

This study used ELK to collect and filter ETL data from the Thai MOOC Platform server, filtering students’ behavioral data for targeted subjects and formatting the data into data frames for simplified k-means learner clustering.

### 3.3 Processes

Following data preprocessing, learners were classified based on their online learning behavior. The k-means clustering algorithm, a robust, unsupervised machine-learning technique, classified data points into distinct clusters. This algorithm was broadly accepted for its effectiveness in classifying students based on their similarities [[42], [43], [44]]. We utilized the classified data to develop predictive AI models using a decision tree algorithm. Both k-means clustering and decision trees are commonly used in educational research due to their simplicity, accuracy, and interpretable results. Therefore, this study uses this algorithm for the preprocessed data. The process involved two steps.

#### 3.3.1 Develop a predictive AI model to categorize online learning behavior

This step creates an AI model that is capable of predicting online learning behavior and classifying students into groups based on this behavior. The model derives its predictive ability from being trained on data from 8000 categorized students, utilizing advanced predictive modeling algorithms.

#### 3.3.2 Classify learners based on online learning behaviors

This step created an AI model that autonomously categorizes learners into groups based on their online learning behaviors. This classification was performed without direct instructor involvement to enhance learner autonomy.

#### 3.3.3 Assess the accuracy of the predictive AI model

At this stage, the decision tree algorithm was used to assess the AI predictive model's accuracy because of its capability of presenting graphic demonstrations of the findings and its precise calculations, which are suitable for predictive model development [44,45].

## 4 Research findings

This section describes the research findings.

### 4.1 Development of a predictive AI model

With reference to research objective 1, this study seeks to develop a predictive AI model to categorize the online learning behavior of Thai students studying with a Thai MOOC. The development phase of the predictive AI model yielded promising outcomes.

#### 4.1.1 Behavioral classification capability

**Fig. 2:**
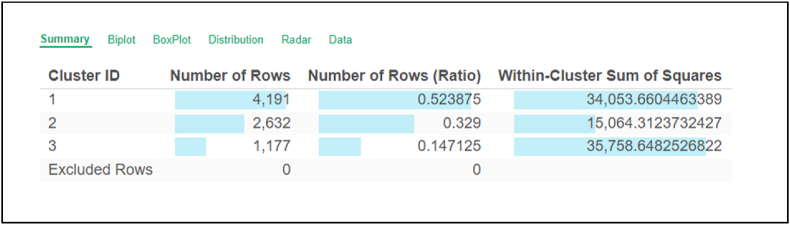
Learners' online learning behavioral classification by AI predictive model using the k-means clustering technique.

K-means clustering successfully grouped learners based on online learning behavior into three categories: the HAP, MAP, and LP groups. Of 8000 participants, 4191 students were grouped into Cluster 1, 2632 into Cluster 2, and 1177 into Cluster 3, as presented in Fig. 2. This classification was used to teach the AI predictive model to classify learners based on their online learning behaviors.

Fig. 2 provides the results of k-means clustering, where students are classified into three groups on the basis of their online learning behavior. The figure also provides the number of students appearing in each cluster.

#### 4.1.2 Predictive capabilities

The developed predictive model accurately predicts learners based on their learning behavior. The results from the development of predictive AI show a high prediction accuracy rate.

#### 4.1.3 Application flexibility

This AI predictive model works automatically, adjusting the data size to fit the model training to increase prediction accuracy. This is useful for both instructors and learners.

### 4.2 Categorizing the online behavior of students enrolled in a Thai MOOC

**Fig. 3:**
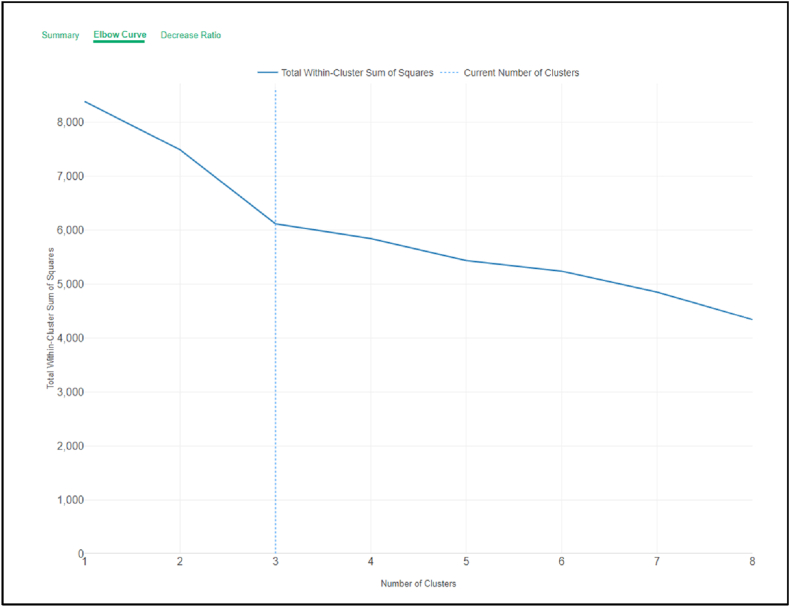
K-value (number of categorized groups).

With reference to research objective 2, this study seeks to categorize the online behavior of students enrolled in a Thai MOOC. The details are as follows:

Before using the k-means algorithm to classify learners’ online behaviors, the elbow technique was used to determine the k-value (number of categorized groups). The results revealed that although the graph illustrated eight clusters in total, the appropriate point for the number of clusters was the point where the graph had the most “elbow” shape, indicating that the number of clusters equals three. Therefore, a k-value of 3 is appropriate for dividing students from this data frame, as illustrated in Fig. 3.

Fig. 3 presents the results of the elbow technique used to determine the appropriate number of clusters for k-means classification in online learning behaviors.

**Fig. 4:**
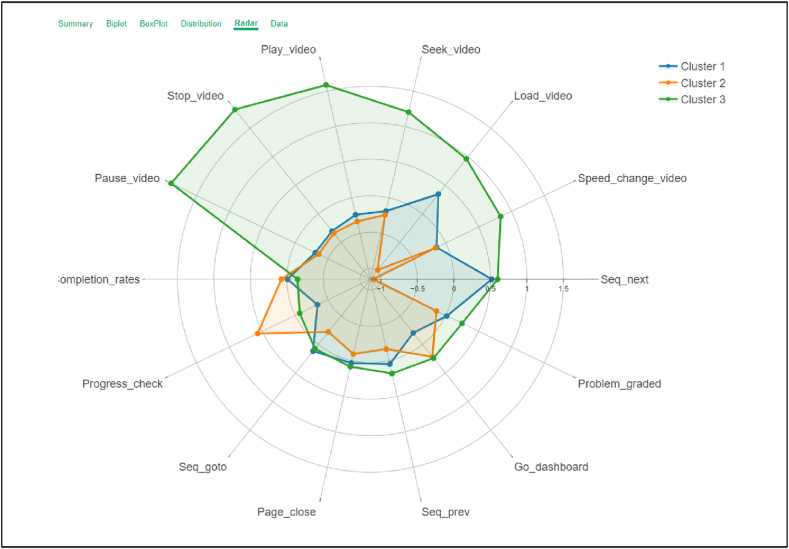
Radar chart of learners' behavior classified by the k-means clustering algorithm.

Categorizing learners according to their online learning characteristics using the k-means clustering algorithm, using ETL data of online learners interacting with the Thai MOOC platform system, drawing on the results of the elbow technique, set the number of clusters to three, is illustrated in the Radar chart in Fig. 4. The chart illustrates the behavior of students interacting with the system in each cluster as follows.

Cluster 1 (Medium Active Participants)

Learners in this group interacted with the Thai MOOC platform at a moderate level and passed the course evaluation criteria. The interaction of these students was moderate in almost all aspects, except accessing courses and checking academic results, which were the lowest of all three groups of students.

Cluster 2 (Lurking Participants)

The students in this group had minimal interaction with the Thai MOOC platform. The low number of clicks on sequential content observed by the Radar chart indicated that they hardly learned the content in the order assigned or did not study it at all. Moreover, they never encountered a video content page. Despite their low interaction with the MOOC platform, the learners in this group had the highest academic success rate and the highest examination scores of the three groups.

Cluster 3 (Highly Active Participants)

Learners in this group interacted highly with the Thai MOOC platform in almost every aspect compared with learners in other groups, especially in navigational and video interaction events. This shows that the students were trying to study in the order the teachers had designed.

Fig. 4 presents a radar chart to visualize learners’ behavior, classified into three clusters using k-means clustering. This chart presents a multidimensional to show how learners in each cluster interact with online learning behaviors.

The classification of online learning behaviors exhibited by Thai MOOC students offers valuable insights for course developers and platform owners. By understanding distinct online learning behavioral clusters, course developers can tailor their courses to more effectively cater to students’ diverse needs. In addition, platform owners can utilize this information to establish effective guidelines for online course development.

### 4.3 Predictive AI Model's accuracy in predicting learners' future behaviors

**Fig. 5:**
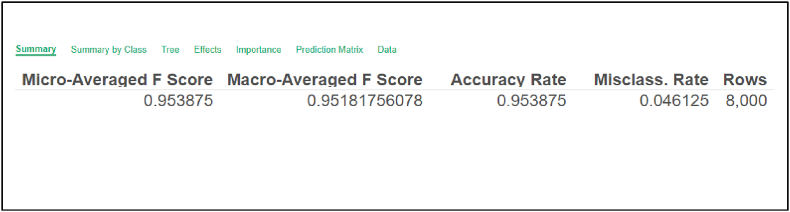
Overall accuracy rate of the AI predictive model.

With regard to research objective 3, this study seeks to evaluate the accuracy of the predictive AI models developed to forecast learners’ online behavior. The details are as follows:

Accuracy evaluation using the decision tree algorithm provides essential insights into the effectiveness of predictive AI models. The reported accuracy rates for decision tree algorithms typically range between 0.89 and 0.98 [29,46,47], The model's overall accuracy rate was 0.953875, reflecting a high level of accuracy, as presented in Fig. 5.

Fig. 5 presents the overall accuracy rate achieved by the AI predictive model using a decision tree algorithm.

**Fig. 6:**
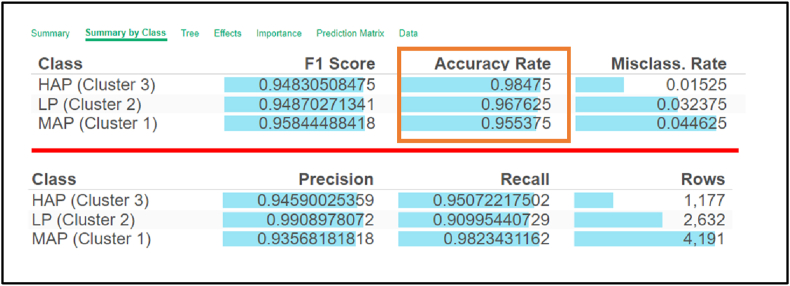
Accuracy rate of the AI predictive model classified by student online behaviors.

The decision tree assessment revealed an 0.98475 accuracy rate of the AI predictive model of the HAP cluster, followed by the LP cluster at 0.967625, and the MAP cluster at 0.955375. All indicated high-accuracy prediction, as presented in Fig. 6.

Fig. 6 presents the accuracy rates achieved by the AI predictive model, using a decision tree algorithm, for three groups of learners, classified based on their online learning behavior.

These accuracy rates indicate that the AI predictive model is proficient in forecasting learners’ future behavior, paving the way for making personalized learning recommendations.

The predictive AI model's accuracy in predicting learners' future behaviors can enable teachers to anticipate how groups of students will interact with the online learning environment. This will help teachers adapt their teaching styles and update the content to better suit the needs of students with diverse characteristics.

## 5 Discussion

This research provides comprehensive insights into the AI-driven system and its ability to categorize learners’ online behavior and predict their future academic success. A primary contribution of this study is the successful development of predictive AI models for classifying learners based on their online behavior without the need for direct instructor involvement.

By implementing this AI predictive model, we found that learners who participated in Thai MOOCs can be classified into three groups: HAPs, MAPs, and LPs. This finding is consistent with the study of [32,33,48] which analyzed and classified students in a Thai MOOC into three groups according to their online learning behavior: active learners, passive learners, and bystanders. However, our findings diverge from studies like [49,50], which identified larger numbers of learner clusters. This discrepancy may reflect variations in data sources and clustering methodologies [38]. analyzed the online behavior of learners who participated in a Thai MOOC using data obtained from interviews and documents and divided learners into three groups: students in the lurking group participate in less than 50 % of the curriculum activities; those in the moderately active group participate in more than 50%–65 % of course activities; and those in the memorably active group participates in more than 65 % of activities.

As a result, using the decision tree algorithm to predict future learners' academic achievement, we achieved high-accuracy rates for the HAP, MAP, and LP learner groups, underscoring the robustness of these systems. This finding is consistent with the study of [51], who used the decision tree algorithm to accurately predict a 96 % dropout rate of MOOC learners. The experiment allows educators to make predictions early in the management process [52]. analyzed the behavior and motivation of online learners using the decision tree algorithm and predicted learner motivations in the MOOC online teaching system into three types: motivation, intrinsic motivation, and external motivation (intrinsic, extrinsic, and motivation) with an accuracy of 75 %. Therefore, the AI predictive model can enhance learner success and retention in online learning environments. Our findings also indicate that AI-driven solutions play a pivotal role: by providing timely interventions and personalized support based on predictive insights, institutions can proactively address learners’ needs and challenges, ultimately leading to improved learning outcomes.

This study's classification of the online behaviors of Thai MOOC students offers valuable insights for course developers and owners of platforms. Tailoring course designs and establishing development guidelines can clarify student needs according to these behavioral clusters. The accurate predictive AI model further empowers teachers. Being able to anticipate student interactions, teachers can adapt their teaching styles and content to better suit diverse learners. An important limitation of this study is that the data were only collected from the KMUTT015 course. To enhance the predictive accuracy of the AI model and potentially discover more nuanced behavioral clusters, future research should employ larger datasets encompassing a wider range of MOOC courses. Moreover, the information available on the ETL on the Thai MOOC platform is not comprehensive due to the limitations in the course design previously mentioned. Access to data from other platforms could potentially reveal a clearer picture of the diversity of behavioral clusters.

## 6 Recommendation

This study develops a successful predictive AI model for classifying learners based on their online learning behavior. The findings shed light on the potential of predictive AI models to revolutionize the landscape of online education in the Thai MOOC platform by categorizing learners based on their learning characteristics and predicting learners' future academic achievement by automating this process without direct instructor involvement. Our predictive AI model empowers learners to navigate online courses at their own pace and style. Future research can apply this procedure to categorize and predict students’ behavior. This study can also offer valuable insights for the effective design of online courses on the Thai MOOC platform and other LMSs. Educational institutions, policymakers, and instructors can leverage the distinct needs of different learner groups to develop courses and guidelines that enhance the quality of online learning experiences.

Future research should explore alternative prediction algorithms or hybrid predictive models to optimize their prediction potential and adapt them to changing data trends. This approach may increase prediction efficiency.

## 7 Conclusions

This study explored the potential of predictive AI models in online learning environments, focusing on the Thai MOOC platform. Our research journey encompassed developing an AI predictive model, classifying online learners participating in Thai MOOCs, and evaluating the system's prediction accuracy.

In our first and second sections, our research demonstrated the successful development of predictive AI models capable of classifying learners into HAP, MAP, and LP groups. This achievement was pivotal in enhancing learner autonomy in online courses, aligning with contemporary educational paradigms emphasizing personalized learning experiences.

In the third section, we evaluated the accuracy of these AI models, which were remarkably accurate across diverse learner groups. These findings substantiated the efficacy of predictive AI models in forecasting learner behavior and performance. Our findings underscore the potential of AI-driven solutions to enhance the quality of online education by offering tailored support to learners at varying levels of engagement.

## Ethical approval statement

Ethical approval was obtained from the ethics committee at King Mongkut's University of Technology Thonburi (Ref No: KMUTT-IRB-COE2019-200).

## Data availability statement

The dataset supporting the findings of this study is available from the corresponding author upon reasonable and justified request and a compiled version of the published data can be accessed at [link]."

## Funding

This research did not receive any specific funding.

## CRediT authorship contribution statement

**Jira Chonraksuk:** Writing – review & editing, Writing – original draft, Visualization, Validation, Supervision, Software, Resources, Project administration, Methodology, Investigation, Formal analysis, Data curation, Conceptualization. **Surapon Bunlue:** Supervision.

## Declaration of competing interest

The authors declare that they have no known competing financial interests or personal relationships that could have appeared to influence the work reported in this paper.
